# The MRI central vein marker; differentiating PPMS from RRMS and ischemic SVD

**DOI:** 10.1212/NXI.0000000000000496

**Published:** 2018-09-26

**Authors:** Amal P.R. Samaraweera, Yasser Falah, Alain Pitiot, Robert A. Dineen, Paul S. Morgan, Nikos Evangelou

**Affiliations:** From the Division of Clinical Neuroscience (A.P.R.S., Y.F., R.A.D., N.E.), University of Nottingham; Laboratory of Image & Data Analysis (A.P.), Ilixa Ltd; National Institute of Health Research (R.A.D.), Nottingham Biomedical Research Centre; and Department of Medical Physics (P.S.M.), Nottingham University Hospitals NHS Trust, Nottingham, United Kingdom.

## Abstract

**Objective:**

To determine whether the assessment of brain white matter lesion (WML) central veins differentiate patients with primary progressive MS (PPMS) from relapsing-remitting MS (RRMS) and ischemic small vessel disease (SVD) using 3T MRI.

**Methods:**

In this cross-sectional study, 71 patients with PPMS, RRMS, and SVD were imaged using a T2*-weighted sequence. Two blinded raters identified the total number of WMLs, proportion of WMLs in periventricular, deep white matter (DWM) and juxtacortical regions, and proportion of WMLs with central veins in all patient groups. The proportions were compared between disease groups, including effect sizes. MS or SVD was categorized using a threshold of ≥40% WMLs with central veins as indicative of MS. Interrater and intrarater reproducibility was calculated.

**Results:**

The mean proportion of WMLs with central veins was 68.4% in PPMS, 74.3% in RRMS, and 4.7% in SVD. The difference in proportions between PPMS and SVD groups was significant (*p* < 0.0005; effect size: 3.8) but not significant between MS subtypes (*p* = 0.3; effect size: 0.29). Distribution of WMLs was similar across both MS groups, but despite SVD patients having more DWM lesions than PPMS patients, proportions of WMLs with central veins remained low (2.75% in SVD; 62.5% in PPMS). Interrater and intrarater reproducibility comparing proportions of WMLs with central veins across all patients was 0.86 and 0.90, respectively. Level of agreement between the proportion of WML central veins and established diagnosis was 0.84 and 0.82 for each rater.

**Conclusions:**

WML central veins could be used to differentiate PPMS from SVD but not between MS subtypes.

Approximately 15% of patients with MS develop progressive symptoms from the onset; primary progressive MS (PPMS)^1,2^ can pose more diagnostic challenges compared to relapsing-remitting MS (RRMS).^3,4^ The presence of white matter lesions (WMLs) becomes more frequent with age,^5^ as such the risk of misdiagnosis of MS rises. Occasionally, the location of WMLs is helpful in distinguishing MS from ischemic small vessel disease (SVD), a common MRI mimic of MS.^6^ Sometimes WMLs in SVD can affect the same brain regions as MS, fulfilling dissemination in space MS MRI criteria.^7^ In SVD, deep white matter (DWM) and subcortical lesions are the most common areas for WMLs,^8^ although periventricular (PV) lesions can also occur.^9^ WMLs in the DWM and juxtacortical (JC) areas are most likely to cause diagnostic confusion between MS and SVD.^10^

T2*-weighted MRI allows visibility of small veins within WMLs secondary to increased deoxyhemoglobin concentrations. This represents the imaging equivalent of inflammatory activity surrounding a vein on histology.^11,12^ Studies in RRMS have shown its potential to differentiate RRMS from neuromyelitis optica^13–15^ and SVD at 3 and 7T.^10,16,17^ Studies at 7T showed PPMS had similar proportions of WMLs with central veins compared to RRMS.^16,18^ We used 3T T2* to (1) determine if the proportion of WMLs with central veins in PPMS is as high as RRMS and (2) determine if the difference between the proportion of WMLs with central veins in PPMS and SVD is as significant as seen in RRMS.

## Methods

### Patient selection

All patients recruited were adults over the age of 18 years who attended the MS or general neurology clinics at Nottingham University Hospitals NHS Trust, United Kingdom with a confirmed diagnosis of PPMS, RRMS, or SVD. Consecutive patients with PPMS seen in the MS clinic were invited for this study if they had at least one brain lesion on their clinical T2 or fluid-attenuated inversion recovery (FLAIR) MRI scan. RRMS patients were part of a wider study looking at the use of central veins in MS. All patients were diagnosed by experienced MS neurologists and were regularly followed up for their MS. All patients had typical MRI findings, supported by a consultant neuroradiology report, with some having CSF testing for oligoclonal bands. Time of initial diagnoses spanned from prior to the year 2000, as a consequence Poser and McDonald criteria 2001–2010 were initially used but all of them fulfill the 2017 criteria.^19^

All patients with a diagnosis of SVD were referred to the neurology service by their general practitioner for a variety of symptoms. The diagnosis was based on clinical evaluation by a consultant neurologist, ruling out symptoms suggestive of demyelination or another white matter disease and identification of vascular risk factors such as a history of hypertension, hypercholesterolemia, diabetes mellitus, smoking, and high body mass index. Some of these patients had supportive laboratory findings (blood tests, e.g., checking cholesterol, autoimmune screen, and sometimes CSF testing to exclude possible demyelination). Each patient had an MRI brain scan reported by a consultant neuroradiologist confirming the appearance of the scan was in keeping with SVD.

Exclusion criteria for both groups included patients who had other autoimmune diseases that could potentially also cause WMLs and patients who were pregnant.

### Standard protocol approvals, registrations, and patient consents

Written informed consent from all patients was obtained before inclusion, and the study was approved by the local research ethics committee.

### MRI protocol

MRI was performed using a 3T Achieva (Philips Healthcare, Best, The Netherlands) with a 32-channel receive-only head coil. The protocol consisted of a high-resolution 3D T2*-weighted gradient echo scan with a high echo planar imaging factor of 15.^20^ The matrix was 448 × 448 × 336 with a non-interpolated voxel size of 0.55 × 0.55 × 0.55 mm. Parallel imaging factors of 2 were used in both phase encoding directions. In addition, the water-only excitation flip angle was 10 degrees, effective echo time 29 ms, repetition time 54 ms, 2 averages, duration 254 seconds.

### Image analysis

Only T2* images were used for the analysis. Images were saved in a DICOM format and then converted to NIfTI format.^21^ Each brain T2* scan was cut into 8 blocks using an in-house algorithm (A.P.). This was done to prevent the brain being seen in its entirety, in an attempt to blind the raters as much as possible to the overall pattern of WMLs with central veins throughout the brain. We aimed to prevent lesion location and the presence of a vein in some lesions influencing the “detection” of veins in other lesions in the same brain, revealing the diagnosis of MS or SVD.

In-house image analysis software NeuROI (nottingham.ac.uk/research/groups/clinicalneurology/neuroi.aspx) was used to identify WMLs manually by raters blinded to the clinical diagnoses. A WML central vein was judged as present if it appeared as a black hypointense line (running along the long axis of the WML) or a hypointense dot within the centre of a WML surrounded by a hyperintense ring. This had to be in at least 2 of 3 orthogonal planes. All WMLs were more than 3 voxels in diameter with demarcated borders. Confluent WMLs with multiple veins coursing through them were not counted, as sometimes it was difficult to identify a definite central vein. Therefore, the vein also had to be located within the centre of the WML irrespective of the lesion shape.^22^ This applied to WMLs in both the MS and the SVD groups. Total WML numbers, WML central vein numbers, and the proportion of WMLs with central veins were calculated for each block of brain volume and total brain volume. The location of each WML in the brain was also assessed by one rater (A.P.R.S.) and classified as (1) PV if one border of the WML was in contact with the ventricular surface; (2) JC if one border of the WML was in contact with cortical grey matter; (3) DWM if the WML did not meet either of the above 2 criteria.^23,24^ Infratentorial lesions were not included in the analysis because of limitations of T2* in detecting lesions in this area (more noise and sometimes it was difficult to delineate lesions from the CSF in between the folia of the cerebellum, which is also hyperintense). Each scan was finally classified as MS if 40% or more of WMLs had central veins or as SVD if less than this. This threshold in previous studies allowed a clear differentiation of the 2 groups.^16,25^

To determine interrater reproducibility of the proportion of WMLs with central veins for each patient (i.e., can each rater determine a similar proportion of WMLs with central veins), a second blinded rater (Y.F.) analyzed 50% of the T2* scans. Additionally to test the intrarater reproducibility, 2 months after the initial assessment, 2 further procedures were followed by blinded assessor (A.P.R.S.); first, 71 randomly selected brain blocks (one from each subject) were assessed for WML number and proportion of WMLs with central veins. Second, 25% of whole brain scans were reviewed again to establish intrarater reproducibility of the whole brain WML assessment and of the disease classification.

### Statistical analysis

Mean values with SDs or medians and interquartile ranges (IQRs) for nonparametric data are quoted. Histograms were used to determine normal and nonnormal distributed data. A Mann-Whitney *U* test was used to compare nonnormal data and an independent *t* test for normally distributed data. An intraclass correlation coefficient (ICC) 2-way random model was used to determine the interrater and intrarater absolute agreement of identifying the proportion of WMLs with central veins. The agreement between 2 blinded raters in terms of the diagnosis (MS or SVD) based on using the presence of WML central veins alone was calculated with a Cohen kappa coefficient, as was the agreement between each rater and the known, established diagnosis. Effect sizes were calculated using Cohen *d*. Statistical significance was set at *p* < 0.05. Statistical analyses were performed using Statistical Package for the Social Sciences (IBM SPSS 22).

### Data availability

All anonymized raw data can be provided by request from any qualified investigator.

## Results

### Demographics

Seventy-one patients were scanned; 32 patients with PPMS (14 female and 18 male patients), 23 with RRMS (13 female and 10 male patients), and 16 with SVD (8 female and 8 male patients). The mean age of the PPMS group was 55.4 ± 9.5 years (range, 35–73 years) and mean expanded disability status scale (EDDS) was 5.6 (range, 2–7); mean age was 41.1 ± 12.3 years (range, 18–64 years) and mean EDSS was 2.7 (range, 0–6) in the RRMS group; mean age was 50.9 ± 9.6 years (range, 38–75 years) in the SVD group. The difference in age between PPMS and SVD patients was not significantly different (*p* = 0.14, effect size: 0.47), although it was between the RRMS and the PPMS groups (*p* < 0.0005, effect size: 1.3). Median disease duration, taken as the time from a confirmed diagnosis by a neurologist to the date of the T2* scan, was 36 months (IQR, 2–144) in the RRMS group and 43.5 months (IQR, 25–63) in the PPMS group (*p* = 0.93).

### PPMS and SVD

**Figure 1:**
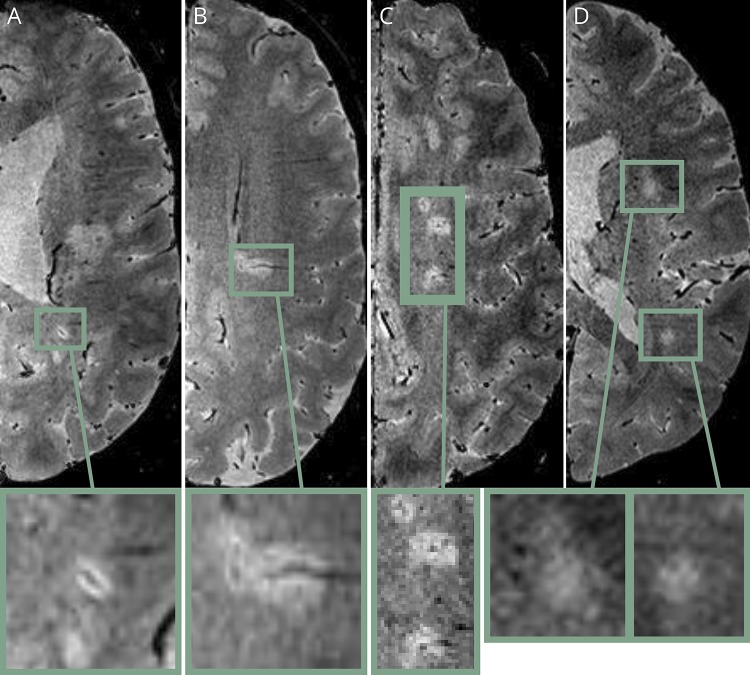
White matter lesion central vein visibility in MS and absence in small vessel disease (SVD) (A and B) Two different patients with primary progressive MS, showing a deep white matter (DWM) lesion with a central vein (A) and periventricular lesion with a central vein (B). (C) A patient with relapsing-remitting MS showing 3 DWM lesions each with a central vein. (D) A patient with SVD showing 2 DWM lesions with no central vein.

**Figure 2:**
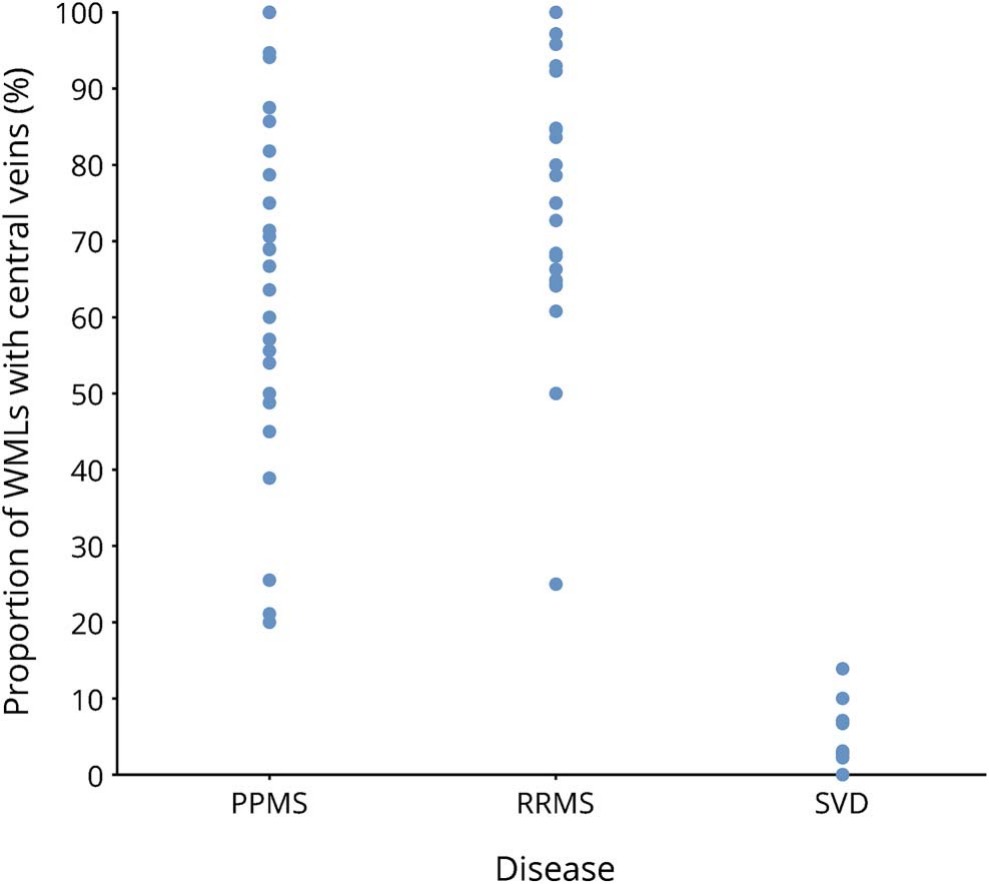
Frequency of the proportion of total WMLs with central veins in PPMS, RRMS, and SVD Results were derived from the analysis of one rater (A.P.R.S.) who analyzed all the blinded scans. PPMS = primary progressive MS; RRMS = relapsing-remitting MS; SVD = small vessel disease; WML = white matter lesion.

Comparisons for these 2 groups are summarized in table 1, with examples of the central vein visibility in PPMS and absence in SVD shown in figure 1. A total of 1,501 WMLs were detected between both groups. SVD patients had a higher number of WMLs (median: 35 [17–44.8]) than PPMS patients (median: 17 [8–44.5]). Although this difference was not statistically significant, there was a trend toward significance (mean difference: −18; 95% CI, −34.5 to −1.5; *p* = 0.06). The mean proportion of WMLs with central veins in the PPMS group was much higher than in those with SVD (table 1 and figure 2).

**Table 1:**
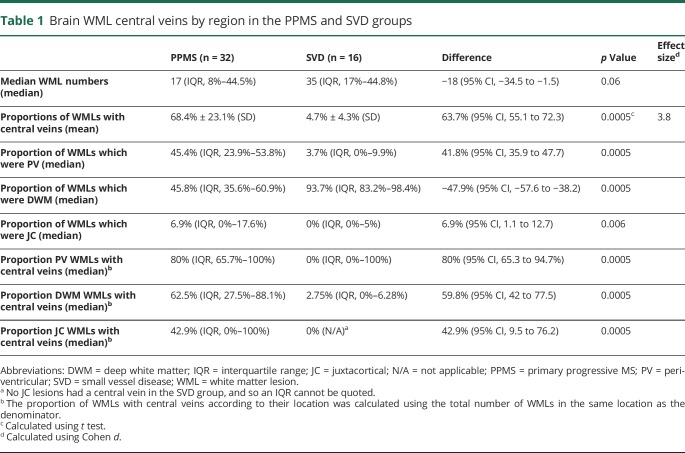
Brain WML central veins by region in the PPMS and SVD groups

### PPMS and RRMS

**Figure 3:**
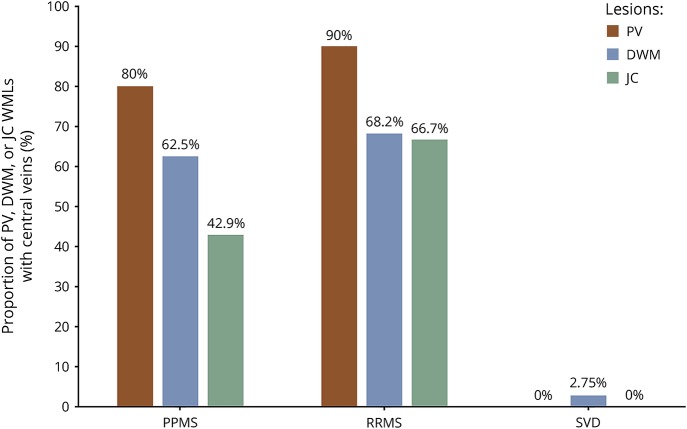
Distribution of WMLs with central veins in the PV, DWM, and JC regions DWM = deep white matter; JC = juxtacortical; PPMS = primary progressive MS; PV = periventricular; RRMS = relapsing-remitting MS; SVD = small vessel disease; WML = white matter lesion.

MRI results for the MS cohorts are summarized in table 2, with examples of the central vein visibility in RRMS shown in figure 1. The total number of WMLs identified in both groups was 1,956. As described in the table, the median WMLs in the PPMS group was lower than in RRMS (*p* = 0.03). The mean proportion of WMLs with central veins in all locations for the PPMS group was similar to the RRMS patients (68.4% and 74.3%, respectively; *p* = 0.3; effect size: 0.29). The differences in proportions of WMLs with central veins in the PV, DWM, and JC regions are summarized in table 2 and figure 3.

**Table 2:**
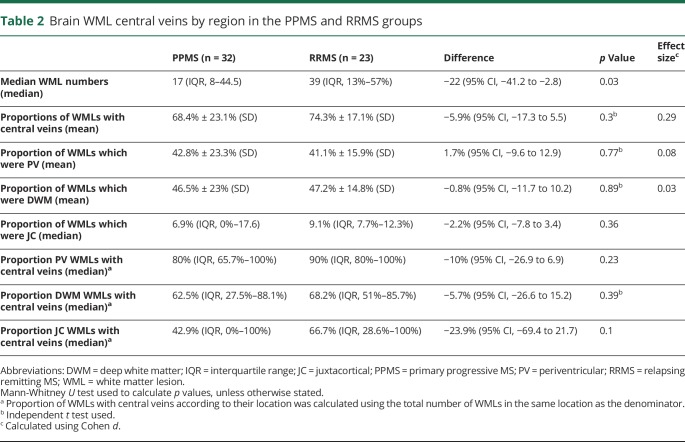
Brain WML central veins by region in the PPMS and RRMS groups

A significant finding was that the proportion of WMLs that were in the DWM region of the SVD group was higher than the PPMS patients.

However, the proportion of these DWM lesions with central veins was much lower, with only 2.75% having central veins in the SVD cohort compared to 62.5% in PPMS (figure 3).

### Interrater reproducibility

Interrater ICC was high when the proportion of WMLs with central veins was compared between both raters (0.86; 95% CI, 0.56–0.94; *p* < 0.0005). The level of agreement between using the proportion of WMLs with central veins alone (≥40% indicative of MS) to categorize a scan as MS or SVD, and the *established* diagnosis was good for both raters (rater Y.F.: 0.84; 95% CI, 0.54–1; *p* < 0.0005 and rater A.P.R.S.: 0.82; 95% CI, 0.67–0.97; *p* < 0.0005).

### Intrarater reproducibility

High values were found for intrarater reproducibility (0.90; 95% CI, 0.73–0.96; *p* < 0.0005).

Intrarater reproducibility was tested again 2 months after the initial assessment by A.P.R. Samaraweera. Seventy-one randomly selected brain blocks (1 from each subject) were assessed for WML number and proportion of WMLs with central veins and compared to the first attempt. For WML numbers, ICC was 0.99 (95% CI, 0.98–0.99, *p* < 0.0005), and for the proportion of WMLs with central veins, ICC was 0.95 (95% CI, 0.91–0.97, *p* < 0.0005).

### Classification of MS or SVD based on 40% threshold of the central vein marker

Testing if both raters agreed with each other about the categorization of MS and SVD for each scan showed moderate agreement (0.54; 95% CI, 0.15–0.93; *p* = 0.001). Twenty-five percent (18/71) of all whole brain scans were reviewed again by one rater (A.P.R.S.), and the categorizations of MS and SVD, and the established diagnoses were compared between both attempts by this rater (0.73; 95% CI, 0.38–1; *p* = 0.001).

## Discussion

We found that WML central veins were present in patients with PPMS using a T2*-weighted sequence at 3T MRI, and hence suggest that the central vein marker could be used as supportive criterion in the diagnosis of PPMS. This has the potential to influence patient care promoting earlier diagnosis of PPMS, with early initiation of treatment in PPMS being shown to be more likely to produce greater benefits.^26^

The new diagnostic criteria for MS^19^ are already advancing early diagnosis in the clinical setting, but they were not developed to differentiate MS from other conditions. PPMS is more difficult to diagnose than RRMS, due to it insidious onset, older age at presentation (and as a consequence greater number of differential diagnoses), less brain WMLs on MRI, and WMLs which can be mistaken for ischemic WMLs.^27^ As a result, patients with a suspicion of progressive MS are more likely to have a longer time from symptom onset to diagnostic confirmation^4^ and have additional investigations, such as spinal cord MRI, CSF testing, and evoked potentials.^28^

Of all the differential MRI diagnoses, distinguishing MS from SVD and other incidental WMLs detected on MRI is one of the most common.^6,29^ This becomes more challenging when the clinical presentation is not with a relapsing pattern, but with progressive neurologic symptoms, as in PPMS. We have found that WML central veins could be used to distinguish PPMS from SVD, as it has been shown with RRMS in multiple studies before.^10,13,14,16,17,25,30,31^ Although SVD patients had more WMLs on average than patients with PPMS, we found that this had no diagnostic value. As expected, the majority of the WMLs in the SVD group were in the DWM, in keeping with the literature on the distribution of lesions in SVD.^10,32–34^ Although this reached statistical significance at the group level, the WML location was not able to differentiate PPMS and SVD (results not shown here). In this study, we specifically evaluated sections of the brain separately, so the observer could not be influenced by the presence of veins in other WMLs of the same brain or overtly be influenced by the location of the WMLs.

The clinically important diagnostic finding of this study was the stark contrast between the proportion of WMLs with central veins in the PPMS group (68.4%) and the SVD patients (4.7%). Even the more common DWM lesions of the SVD group had fewer central veins compared to DWM lesions of PPMS patients (approximately 3% vs 62%).

The central vein marker has been found to be diagnostically useful in MS before; however, most studies have reported on RRMS patients that usually pose less of a diagnostic challenge than PPMS. Until now, to our knowledge, 2 studies have reported on a small number of PPMS patients with T2*-weighted imaging at 3T.^17,35^ Our study confirmed both the pathologic findings of perivenous demyelination in PPMS, and ultra-high-field MRI results.^16,18^

We found that all PPMS and RRMS patients studied had similarly high proportions of WMLs with central veins, and higher than all individuals with SVD. This has been shown in smaller cohorts in previous studies only at 7T.^18^ Like previous reports, our work has not been able to differentiate the 2 MS subtypes apart using the central vein marker, which is not surprising considering the histopathologic similarities of the 2 MS subtypes. Although the number of WMLs was, as a group, lower in the PPMS patients, the location of WMLs in the supratentorial brain was similar across both MS groups, with most WMLs in the PV and DWM regions. WMLs in the PV region had high numbers of central veins (80% of PV lesions in PPMS and 90% in RRMS). This would be expected because of the high venous density in the PV distribution, caused by deep medullary veins draining toward subependymal veins of the lateral ventricles.^36^ Furthermore, over 60% of DWM lesions in both MS groups also had central veins. Any small differences in the proportions of PV, DWM, and JC lesions with central veins between PPMS and RRMS patients were not significant, reinforcing that lesion location and the presence of central veins cannot distinguish the 2 subtypes. This also is in keeping with the evidence suggesting similarities in the pathophysiology of WML formation in these 2 subtypes of MS.

The diagnostic rule of using 40% of WMLs with central veins as a cut-off for categorizing MS allowed high agreement with the established diagnoses for both raters. However, moderate agreement was shown when using this rule to determine if both raters agreed with each other about the established diagnosis. We can only hypothesize that identifying all WMLs and central veins may lead to more error (potentially mistaking CSF, perivascular spaces, nonspecific lesions, and cortex as demyelinating lesions). More studies will be needed to assess if identifying a subset of WMLs with central veins is more accurate.^31^

Our study cohorts were derived from a typical UK neuroscience centre with a large outpatient clinical setting, combining both general neurology and MS clinics. The demographics were as anticipated. The mean age of our RRMS cohort was lower than the PPMS group, with slightly more females than males in the RRMS group and the opposite in the PPMS cohort. Our PPMS patients had a higher level of disability compared to RRMS patients. WML numbers were also lower in the PPMS group in keeping with the previous literature.^1,37^

Some limitations of this study should be noted. First, there was a difference in age between the PPMS and RRMS group, with a mean difference of 14.2 years. As nonspecific WMLs are more commonly seen with advancing age, one would have expected a higher number of non-MS WMLs without a central vein in the older PPMS group.^32^ However, the difference in the proportion of WMLs with central veins between PPMS and RRMS groups was not statistically different. Infratentorial WMLs were also not analyzed as we detected very few lesions in this region using the T2* sequence (3 in PPMS, 2 in RRMS, and 3 in SVD groups). This is possibly a limitation of T2* in this location. Despite this, as the proportion of WMLs with central veins in the supratentorial brain was high for both MS groups, the exclusion of a few infratentorial lesions probably would not affect the overall ability to differentiate MS from SVD. Additionally, our study did not use the central vein marker in patients with PPMS or SVD at disease onset. A prospective study in the difficult to diagnose phenotype needs to validate our cross-sectional central vein data. This is currently under way at our institution. Furthermore, if the central vein marker ever entered clinical practice, training for clinicians reporting scans would be needed if, for example, the North American Imaging in Multiple Sclerosis consensus criteria^22^ are used.

A number of centers have reported that fused images (e.g., FLAIR*) qualitatively can detect infratentorial lesions with central veins reliably.^35,38^ Similarly, some advocate the use of contrast agents in the detection of the central vein. No doubt over the next few years, more specific MRI sequences and refinement of the central vein criteria will be developed optimizing the use of this new MRI sign. We would expect similar or larger differences between the MS and non-MS groups of patients with any improved methods.

Our findings show that WML central veins are present in PPMS patients in as high proportions as those found in RRMS. These can be identified in WMLs irrespective of the chronicity of the disease using 3T, noncontrast T2*-weighted imaging. The difference in these proportions between PPMS and SVD patients is significant, irrespective of supratentorial brain location, and may be helpful in the clinical setting when there is difficulty in differentiating these 2 conditions, clinically or radiologically.

In this article, we wanted to demonstrate the clear potential of the WML central vein marker in the differentiation of PPMS from SVD using clinical scanners. The exact methodology used by neuroradiologists in clinical practice will depend on the gradually accumulating clinical experience, as the central vein marker is increasingly being used by different centers. Specific prospective studies assessing the minimum number of WMLs required to be assessed in patients with diagnostic uncertainty need to be undertaken. Additionally, studies comparing the diagnostic benefit of the central vein marker to the recent 2017 McDonald criteria would be important to perform, alongside comparing its use to other current diagnostic tests, e.g., CSF oligoclonal bands, spinal cord inflammatory lesions and using a higher number of WMLs for dissemination in space.

Using noncontrast T2* imaging and clinical 3T MRI scanners, present now in many large hospitals worldwide, we can detect the central vein marker in much higher proportions in patients with PPMS and RRMS compared to patients with SVD. This could be diagnostically useful. The new MS diagnostic criteria strengthen the value of lumbar punctures in the diagnosis of MS.^19^ Not all patients of course are keen or willing to have a lumbar puncture. One can speculate that the WML central vein marker might possibly offer a noninvasive alternative to lumbar puncture if the diagnosis is in doubt. That of course would require confirmation by a specific study.

## Glossary

DWM: deep white matter
ICC: intraclass correlation coefficient
IQR: interquartile range
JC: juxtacortical
PPMS: primary progressive MS
PV: periventricular
RRMS: relapsing-remitting MS
SVD: small vessel disease
WML: white matter lesion
